# Neuroinflammation as a Therapeutic Target in Retinitis Pigmentosa and Quercetin as Its Potential Modulator

**DOI:** 10.3390/pharmaceutics13111935

**Published:** 2021-11-16

**Authors:** Joseph Thomas Ortega, Beata Jastrzebska

**Affiliations:** Department of Pharmacology, School of Medicine, Cleveland Center for Membrane and Structural Biology, Case Western Reserve University, 10900 Euclid Ave., Cleveland, OH 44106, USA; jto22@case.edu

**Keywords:** flavonoid, inflammation, photoreceptor, retinitis pigmentosa, rhodopsin

## Abstract

The retina is a multilayer neuronal tissue located in the back of the eye that transduces the environmental light into a neural impulse. Many eye diseases caused by endogenous or exogenous harm lead to retina degeneration with neuroinflammation being a major hallmark of these pathologies. One of the most prevalent retinopathies is retinitis pigmentosa (RP), a clinically and genetically heterogeneous hereditary disorder that causes a decline in vision and eventually blindness. Most RP cases are related to mutations in the rod visual receptor, rhodopsin. The mutant protein triggers inflammatory reactions resulting in the activation of microglia to clear degenerating photoreceptor cells. However, sustained insult caused by the abnormal genetic background exacerbates the inflammatory response and increases oxidative stress in the retina, leading to a decline in rod photoreceptors followed by cone photoreceptors. Thus, inhibition of inflammation in RP has received attention and has been explored as a potential therapeutic strategy. However, pharmacological modulation of the retinal inflammatory response in combination with rhodopsin small molecule chaperones would likely be a more advantageous therapeutic approach to combat RP. Flavonoids, which exhibit antioxidant and anti-inflammatory properties, and modulate the stability and folding of rod opsin, could be a valid option in developing treatment strategies against RP.

## 1. Introduction

Inflammation is an evolutionarily conserved response by the immune system to harmful stimuli such as pathogens, toxins, and tissue damage [1,2]. The principal function of inflammation is to localize and minimize the damage to restore tissue homeostasis. A temporary and controlled upregulation of inflammatory mediators occurs during the normal (acute) inflammatory response. Although the inflammatory response is tissue-specific and depends on the nature of the initial stimulus, the common mechanisms involved in inflammatory response include (1) recognition of the detrimental signal by cell surface receptors such as Toll-like receptors (TLRs); (2) activation of intracellular inflammatory pathways, including NF-κB, MAPK, and JAK-STAT pathways; (3) release of inflammatory markers such as cytokines and chemokines; and (4) an increase in the migration of inflammatory cells such as neutrophils and eosinophils. The acute inflammatory response can become chronic under prolonged insult [3,4,5]. Chronic inflammation is a common pathogenic marker of various chronic diseases like cardiovascular disease, diabetes, arthritis, Alzheimer’s disease, cancer, and ocular diseases, including retinitis pigmentosa (RP) among others [6,7,8]. Tissue-resident macrophages are involved in immune defense in addition to other diverse roles they have, such as regulation of metabolic function, clearance of cellular debris, and tissue remodeling [9]. Macrophages can be polarized into different functional phenotypes depending on their origin and tissue microenvironment. Microglial cells are the macrophages resident in the brain and the retina [4,10]. Microglia function as a checkpoint for the immune system as they express the receptors recognizing the pathogen-associated molecular patterns and harmful factors generated as a consequence of tissue injury [11,12]. In the retina, microglia have been recognized as a pivotal factor in maintaining eye homeostasis [13]. Upon injury, activated microglia induce a robust response of the innate immune system leading to the production of pro-inflammatory mediators and triggering the activation of adaptive immunity. Although activation of microglia is essential to repair the injured tissue, their uncontrolled inflammatory responses contribute to the severity of many degenerative diseases. Microglia activation is classified into two states: (i) M1, an activated or pro-inflammatory neurotoxic state, characterized by the production of inflammatory cytokines such as tumor necrosis factor (TNF)-α, interleukin (IL)-6, and IL-1β; and (ii) M2, an anti-inflammatory or neuroprotective state. The M2 state leads to an increase in the production of some well-characterized markers such as anti-inflammatory cytokines (IL-1 receptor antagonist, IL-4, transforming growth factor (TGF)-β, and IL-10). The M2 state is also associated with an increase in ARG1, an enzyme related to arginine metabolism and wound healing. All these mediators are associated with the decrease of inflammatory cells, an increase in the extracellular matrix protecting proteins for wound repair, and elevation of phagocytosis-associated receptors, such as scavenger receptors [14,15,16]. Microglia polarization states change in the course of inflammation and the disease. However, there are no precise boundaries defining these changes. Thus, microglia plasticity and its role in the inflammatory response in neurodegenerative diseases is a vivid scientific field open for investigation to gain a deeper understanding of these processes.

## 2. Inflammatory Processes in Retinal Diseases

The transduction of environmental light to neural signals in the brain demands unique metabolic and physiological conditions and is carried out by sensory neurons located in the retina. The retina is a highly organized multilayered tissue, composed of many distinct retinal cell types, which provide essential metabolites, phagocytose waste, and control the homeostasis of the surrounding microenvironment [17]. Three types of resident retinal glial cells such as Mϋller glial cells, astrocytes, and microglia, support the retina’s structural integrity and homeostasis. Mϋller glial, the most common glial cells in the retina, span across the entire thickness of this tissue, while microglia reside in the plexiform layer under normal physiological conditions. However, in pathological conditions, microglia cells migrate to the region of injury and serve as an initial host defense system [17]. Under acute insult, microglia mediate neuroprotection and trigger regenerative processes to preserve retinal health. However, under persistent insults such as inherited mutations, prolonged oxidative stress, or hypoxia, the inflammatory response becomes dysregulated and can aggravate tissue damage [12,18,19]. Thus, the retinal microglia can have a dual function: a beneficial role in the homeostatic state and a detrimental effect in a disease state caused by a chronic pathogenic condition. They provide either neurotrophic support or exacerbate neuroinflammation in response to injury. In the second scenario, several changes occur in the microglia, including changes in these cells’ morphology, function, and up-regulation of the expression and secretion of inflammatory markers. Dysregulated innate immune responses in the eye play an important role in the pathogenesis of retinal degenerative diseases, including age-related macular degeneration (AMD), RP, diabetic retinopathy, and glaucoma [19,20]. Thus, understanding the mechanisms related to cellular and molecular events in the inflammatory processes and recognizing the specific markers involved in these processes may support the discovery of new therapeutic targets to alleviate the progression of retinal cell death, preventing vision loss. This review will focus on inflammation and strategies to suppress the inflammatory responses to protect retinal cells in retinopathies caused by genetic disorders such as RP.

## 3. Retinitis Pigmentosa

### 3.1. Epidemiology

Retinitis pigmentosa (RP) is a clinically and genetically heterogeneous hereditary disorder causing progressive retinal degeneration that leads to a decline in vision and eventually blindness (https://rarediseases.org/rare-diseases/retinitis-pigmentosa/ (accessed on 2 November 2021). Mutations in more than 70 genes expressed predominantly in photoreceptor cells and retinal pigment epithelium (RPE) cells, which are related to phototransduction, retinoid cycle, and maintenance of photoreceptors, can cause RP [21]. These mutations can be inherited as autosomal recessive, autosomal dominant, or X-linked recessive traits. RP is one of the most prevalent retinal degenerative diseases. Over 2 million people in the world and about 100,000 people in the United States suffer from blindness caused by RP (https://rarediseases.org/rare-diseases/retinitis-pigmentosa/ (accessed on 2 November 2021). Unfortunately, therapeutic options for RP are limited, stressing the need for the development of new treatment strategies to prolong the visual perception in RP patients.

### 3.2. Pathophysiology

The primary cause of retinal degeneration in RP is the death of rod photoreceptors caused by the conformational aberration in the protein structure due to the genetic change followed by the secondary death of the neighboring cone photoreceptors [22]. Initially, patients experience a decline in dim light vision and loss of peripheral vision. Central vision and consequently daylight vision loss occur at the later stage of the diseases as a result of cone photoreceptor degeneration. Due to the progressive deterioration of photoreceptors, the reorganization of retinal structures that involves phagocytic activity of glial cells is required. During the early onset of degenerative processes in RP, Mϋller glia mediate phagocytosis of declining rods to mitigate retinal damage. These retina resident glial cells do not require migration and are capable of quickly engulfing apoptotic cell bodies in the initial phase of rod photoreceptor degeneration. Eventually, microglial cells become activated either due to signals released from dying rods or crosstalk with Mϋller glia and migrate to the outer retina where they contribute to phagocytic activities [23,24]. These reactive microglia secrete high levels of pro-inflammatory cytokines and chemokines that in conjunction with other factors like oxidative stress, unfolded protein response (UPR), and/or changes in the expression of genes involved in cellular metabolism compromise the viability of cone photoreceptors regardless of the genetic impairment [25,26,27]. Despite the increasing knowledge on RP pathogenesis the underlying mechanism of photoreceptor degeneration in RP is not fully understood and requires further comprehensive examination. However, it is known that several cellular and biochemical processes contribute to photoreceptor death. These processes are described below in more detail.

#### 3.2.1. Endoplasmic Reticulum Stress and Unfolded Protein Response in RP

The endoplasmic reticulum (ER) is a membranous network responsible for translation, folding, and maturation of newly synthesized proteins before their transport through the Golgi structures to their destination. Aberrant mutations often abrupt the proper folding of the polypeptide chain and lead to the accumulation of misfolded protein within the ER [22,28]. To restore ER homeostasis in such a scenario the eukaryotic organisms developed an adaptive mechanism called the unfolded protein response (UPR), a collection of signaling pathways that aims to clear the unfolded proteins [28]. Three main signaling proteins that reside in the ER membranes and their related pathways are involved in the UPR, namely IRE1α (inositol-requiring protein-1α), PERK (protein kinase RNA (PKR)-like ER kinase), and ATF6 (activating transcription factor 6) [29,30]. Under normal physiological conditions, these proteins are inhibited by the residential chaperone binding immunoglobulin protein (BiP). However, under the ER stress caused by the aggregated unfolded proteins, BiP is induced to assist the correct folding. In addition, PERK, IRE1α, and ATF6 become activated, which starts the cascade of signaling reactions to alleviate the over-accumulation of misfolded proteins in the ER by inducing protein degradation mechanisms [28]. Selective activation of IRE1α and ATF6 pathways reduces the levels of multiple misfolding rhodopsin mutants including P23H, T17M, Y178C, C185R, D190G, and K296E without affecting the levels of WT rhodopsin [29,30]. However, activation of PERK leads to unspecific degradation of both the mutant and WT rhodopsin [30]. Thus, only IRE1α and ATF6, but not PERK pathways, could be targeted in efforts to develop therapeutic strategies against RP.

#### 3.2.2. Oxidative Stress in RP

The retinal tissue is at risk of increased oxidative stress due to the high metabolic rate in the retinal cells required for the efficient signal transduction and metabolite turnover to sustain vision [17]. In such a scenario, a fine balance between the oxidative species and antioxidant mechanisms is necessary to maintain cellular homeostasis. However, in pathological conditions such as RP, the efficiency of the homeostatic mechanisms to counter oxidative stress often declines, disrupting the balance between pro- and antioxidative signaling, leading to excessive oxidative stress, inflammation, and apoptosis [31,32]. The majority of reactive oxygen species (ROS) with a predominant singlet oxygen superoxide O_2_^−^ are produced in the mitochondria during respiratory processes. However, the other cellular components, including enzymes located in the ER or plasma membrane also contribute to ROS generation [33,34]. These metabolic reactions generate the oxidant hydrogen peroxide H_2_O_2_, which can facilitate the formation of more toxic-free hydroxyl radical OH֗. Under normal physiological conditions, ROS act as mediators of cellular signaling and are neutralized by the antioxidant defense system, including glutathione (GSH) peroxidase, and superoxide dismutase (SOD) enzymes [35]. However, an imbalance between antioxidant defense mechanisms and ROS production within the cell causes oxidative stress. The excessive free radicals that accumulate within the cell modify the cellular components, including lipids, proteins, and DNA [36]. Thus, oxidative stress is linked to the progression of neurodegenerative diseases, including RP. As mentioned before, in the retina, the oxygen level is high to support the metabolic demands required for signal transduction and quick turnover of the visual chromophore to continuously support the visual processes. During the progressive photoreceptor degeneration in RP, the use of oxygen drops due to decreasing numbers of rod photoreceptors. Consequently, the oxygen level in the retina increases, which enhances the formation of ROS and toxic free radicals. Furthermore, elevated ROS cause oxidative stress to the remaining rods and cones, which accelerates retinal degeneration [37]. ROS also produce detrimental effects on the RPE cells by damaging their lysosomes, which results in a decrease in the RPE phagocytic capacity to degrade the photoreceptor outer segment material [38]. This decrease in the lysosomal activity has been associated with retinal degenerative diseases such as AMD, diabetic retinopathy, and RP [39,40,41]. Oxidative stress has been noted as an important chronic stressor contributing to retinal damage in patients with RP. Indeed, recent studies have discovered that exposure to oxidative stress determines the altered expression of micro-RNA and long non-coding RNA that is likely implicated in the pathogenesis and progression of RP [42].

#### 3.2.3. Inflammation in RP

The retina is a part of the central nervous system (CNS), which translates the image into the electrical neural impulse in the brain. In the retina, the neuroinflammatory response to a pathogen occurs similarly to that in the brain and primarily involves the activation of microglial cells [43,44]. In addition, the retina-specific Mϋller glial cells are involved in the retinal inflammatory response [23,24]. In RP, along with the ER stress-mediated activation of the UPR pathways triggered by the aberrant genetic background, the endogenous molecules released from degenerating photoreceptors induce the innate immune cells, resulting in the activation of the inflammatory response. The key players in retinal inflammation are the microglia activated to exert neuroprotection for degenerating photoreceptors [12]. The anti-inflammatory cytokine TGF-β induces a protective effect for deteriorating photoreceptors during the early stage of RP, which is mediated by microglia signaling [45]. However, due to the presence of the abnormal gene, the continuous activation of microglia results in dysregulated expression and secretion of pro-inflammatory markers, which eventually lead to cellular and tissue damage [46,47]. Indeed, as previously reported, increased levels of several pro-inflammatory cytokines, including IL-1β and IL-6, and an upsurge in the phagocytic activity of the microglia, were found in vitreal samples obtained from humans affected by RP [48,49].

To gain a better understanding of the molecular processes that occur in the human retina in RP, studies using animal models are critical. The most common mouse lines used to study pathophysiology in RP are retinal degeneration (rd)1 and rd10 mice that carry nonsense or missense mutation in the β-subunit of cGMP phosphodiesterase gene (*Pde6b*), respectively [50,51]. In addition, rat and mouse models that carry a mutation in the *Rho* gene, especially P23H, are frequently used to study the mechanisms of RP [52,53,54]. The hallmark of rd1 mice is early-onset retinal degeneration with a single layer of photoreceptors left by 4 weeks of age, while rd10 mice display slower retinal degeneration [50,51]. The microglia infiltration to the retinal photoreceptors layer was observed in rd1 mice at postnatal (P) day 14 and in rd10 mice at P21, indicating their role in the early stages of rod photoreceptor deterioration. A transcriptome profiling study in rd10 mice revealed enhanced expression of cytokines IL-1β, IL-6 and TNF-α, chemokines CCL3 and CCL5, as well as markers of glial regulatory pathways [55]. In addition, it has been suggested that the retinal Mϋller glial cells guide the migration of the microglia and macrophages to the outer retina to clear dying photoreceptors through the release of cytokines and other inflammatory markers [23,56]. In fact, in the RP-mimicking retina degeneration induced in rats by N-methyl-N-nitrosourea (MNU), the Mϋller glia enhanced the secretion of Cx3cl1 cytokine, which induced an increase in Cx3cl1 levels in microglia and triggered their migration to the outer retina [57]. Depletion of Cx3cl1-positive microglia in rd10 mice led to changes in phagocytic activities of these cells and the removal of dying photoreceptors [25,58]. In both rd1 and rd10 mice, uncontrolled secretion of pro-inflammatory chemokines CCL2 and TNF-α by the activated microglia exacerbated the severity of the disease [59]. Moreover, TNF-α induces the NF-κB signaling pathway and leads to upregulation of the NOD-like receptor protein 3 (NLRP3) inflammasome [60]. An increased level of TNF-α, upregulation of the NF-κB, and NLRP3 expression throughout the retina were also reported in another RP-linked Q344X rhodopsin mouse model [61]. Furthermore, an increase in death signaling molecules such as phosphatidylserine within the membranes of dying rod photoreceptors stimulates phagocytosis and further increases the activation of microglia and macrophages via TLR4. The degeneration of rod photoreceptors in RP is mediated by several death processes, including apoptosis, necrosis, and pyroptosis [62,63]. The last is activated through the inflammasome NLR protein family, an adaptor protein called apoptosis speck-like protein (ASC), and caspase 1. The canonical inflammasome promotes the activation of the cytokines IL-1β and IL-18, boosting the infiltration of more immune cells to the retina, resulting in the upkeep of the inflammatory state, which ultimately leads to pyroptotic cell death [64]. In addition, cyclooxygenase (COX)-1, an enzyme involved in the synthesis of prostaglandins, which is highly expressed in the microglia, has emerged as a pivotal player in neuroinflammation in the CNS [64]. Upregulation of COX-1 was found in several models of neurodegenerative disorders [65,66]. As reported, deletion of the *Cox-1* gene or pharmacological inhibition of this enzyme significantly reduced inflammation and enhanced survival of photoreceptors in rd10 mice evidenced by the improved visual function in the retina [66]. Moreover, inhibition of the prostaglandin E2 (PGE2) EP2 receptor also delayed photoreceptor degeneration in rd10 mice [66]. Thus, the COX-1/PGE2/EP2 signaling pathway plays a major role in neuroinflammation onset and retina degeneration in rd10 mice. In addition, recent studies indicate that non-ocular systemic inflammatory processes contribute to the progression of retinal degenerative disorders. This observation emerged from a study deciphering the consequences of lipopolysaccharide (LPS)-induced systemic inflammation performed in the P23H rhodopsin rat model [67]. Systemic injection of LPS into these rats resulted in an enhanced decline in the visual responses, which was associated with increased deterioration of photoreceptor cells. These symptoms were accompanied by an increased number of activated microglia cells and upregulated expression of inflammatory markers and apoptosis-related genes. Thus, chronic exacerbation of the inflammatory response by LPS accelerated the retinal degeneration in the RP-linked P23H rhodopsin rats. These results encourage the pursuit of further in vivo studies evaluating the effect of systemic inflammation in ocular neurodegenerative diseases.

**Figure 1:**
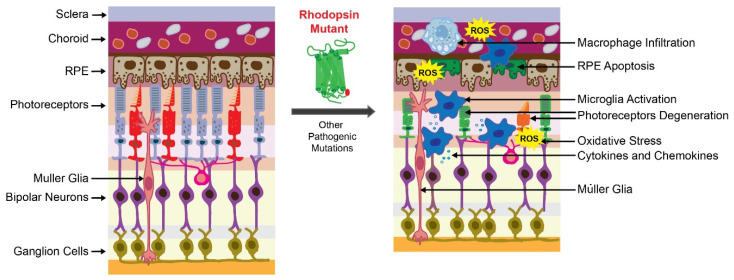
Retinal degeneration in retinitis pigmentosa (RP). The scheme of the healthy retina is shown on the left. The scheme of the degenerated retina caused by the RP-related mutation is shown on the right. The presence of the mutant receptor triggers degeneration of photoreceptors and retinal pigment epithelium (RPE) cells that are critical for retinal homeostasis. In RP, a genetic insult that causes protein misfolding is associated with the activation of cellular unfolded protein response (UPR) and overproduction of reactive oxygen species (ROS), leading to oxidative stress in the retina. Altogether, these factors activate the inflammatory response orchestrated by microglia and macrophages to clear dying photoreceptors. These immune cells release inflammatory mediators that become dysregulated under persistent insult related to the mutant receptor and increase retinal damage. In addition, mutations in other genes related to phototransduction, retinoid cycle, and maintenance of photoreceptors can cause RP.

Together, RP pathological upset is related to the activation of the microglia inflammatory response through inflammasome and releasing of inflammatory substances that contribute to the degeneration of rod photoreceptors followed by the death of cone photoreceptors (Figure 1). These degenerative processes lead to the loss of central vision and eventually total blindness. In addition, the progression of retinal degeneration in RP could be accelerated by systemic inflammatory processes.

## 4. Pharmacological Management of Inflammation in RP

Clinical and experimental studies indicate that inflammation plays an important role in the onset of retinal degeneration and vision loss in RP. Thus, targeting the inflammatory response could potentially result in developing treatment strategies for RP independently of the genetic background. This therapeutic approach focuses on the molecular mechanisms that occur outside of the rods with an idea of blocking signaling pathways that potentiate the photoreceptor degeneration to revert, block, or slow down degenerative processes occurring in photoreceptors.

### Modulation of Microglia-Related Inflammation in RP

The inhibition of microglia activation, blocking chemokine receptors, and decreasing or inhibiting inflammatory mediators are potential molecular targets in RP. Targeting the inflammatory response with dexamethasone, a synthetic anti-inflammatory steroid, decreased microglia activation in rd10 mice evidenced by the lower expression of pro-inflammatory chemokines, which consequently resulted in the preservation of cones and cone-mediated vision [68]. Microglia activation in rd10 mice could also be suppressed by minocycline, a semisynthetic tetracycline derivative. This treatment improved retinal structure and function and prolonged the survival of photoreceptor cells in these mice [69]. Targeting the receptor for TNF-α, an inflammatory factor released by activated microglia to inhibit inflammation was also evaluated. Treatment with an antagonist of the TNF-α receptor such as infliximab and adalimumab resulted in improved retinal health in rd10 mice [70]. Administration of these inhibitors reduced activation of microglia and the NLRP3 inflammasome. Inhibition of inflammatory processes was also achieved by targeting myeloid differentiation factor 88 (MyD88), which is an adaptor protein for the IL-1β and TLRs [71]. MyD88 mediates the inflammatory response to cellular injury by activating NF-κB [72]. The blockage of MyD88 by a specific peptide increased the function of rod photoreceptors and reduced apoptosis in rd10 mice as compared to non-treated controls, suggesting that MyD88 promotes the migration of microglia/macrophages within the retina to the site of injury. Together, several different strategies targeting inflammation in RP proved to help in slowing down the progression of this retinopathy.

## 5. Therapeutic Potential of Polyphenolic Compounds against RP

The use of plants or plant extracts to prevent or treat various diseases has a long history. Natural products are an important source of biologically active compounds and they play a key role in the development of new lead compounds [73,74]. The natural dietary polyphenol compounds, ubiquitously present in fruits and vegetables, are related to a broad range of medicinal properties such as antioxidant, anti-inflammatory, antibacterial, antiviral, and neuroprotective effects [74,75,76,77]. The wide chemical diversity of these plant secondary metabolites, including modification such as glycosylation, increases their biological activity and regulatory effects in multiple cellular pathways at the molecular level [78,79,80]. In addition, polyphenolic compounds such as flavonoids also can modulate the structural properties of the visual receptor, rhodopsin [81,82]. Growing evidence indicates the beneficial effects of bioactive polyphenolic compounds in ameliorating degeneration in ocular diseases, including RP [83,84,85]. As reported previously, the treatment of P23H-1 rats with curcumin at a dose of 100 mg/kg daily between P30 and P70 reduced aggregations of mutant rhodopsin and enhanced its routing to the outer segments, resulting in the improvement of retinal morphology and function [86]. Consequently, the levels of ER stress markers were also decreased in the treated animals. In addition, P23H-3 rats administered with safranal, the main component of saffron, at a dose of 400 mg/kg body weight twice a week for four months displayed slower retinal degeneration in comparison to the vehicle-treated control rats. The treatment with safranal also prevented secondary degeneration of cone photoreceptors [87]. In addition, the recent in vivo studies performed in rd10 mice demonstrated the beneficial effects of two common flavonoids, naringenin and quercetin, in slowing down the progression of cone cell death in RP [88]. These positive effects of polyphenolic compounds in eye-related diseases are likely associated with their antioxidant and anti-inflammatory properties. However, the direct modulatory effect on RP-associated rhodopsin mutants cannot be excluded, as we found positive modulatory effects of flavonoids on the stability of rod opsin and folding of RP-linked P23H rod opsin mutant in vitro [81]. In the following part of this review, we will focus on the beneficial properties of quercetin in retinal degeneration.

### 5.1. Quercetin

The bioflavonoid quercetin has a wide spectrum of biological activities. There are many quercetin derivatives with a broad chemical diversity, occurring naturally in foods. Quercetin has received the most attention in this regard, and its protective effects have been extensively investigated in various in vitro and in vivo models of retina degeneration.

#### 5.1.1. Quercetin Structure and Distribution

Quercetin, the 3,3′,4′,5,7-pentahydroxyflavone, is a common flavonoid that possesses two pharmacophores, the catechol group in the B ring and the OH group at position 3, important for its biological activities. Quercetin is present in fruits and vegetables with berries, peppers, onions, red apples, and broccoli being especially rich in this flavonoid. The average daily intake of quercetin is about 10 mg, but it can achieve up to 100 mg/day depending on the consumption. In foods, quercetin is present mostly in glycosylated form. However, aglycone quercetin supplements are commercially available. These supplements are well tolerated with no significant side effects at the recommended dosage.

#### 5.1.2. Pharmacological Properties of Quercetin

Flavonoids are relatively poorly soluble in aqueous solution and quite unstable in the acidic environment of the stomach, which affects their bioavailability. However, flavonoid metabolism highly depends on the chemical nature of the compound. The solubility of quercetin is about 60 mg/L at 16 °C; however, it solubilizes better in organic solvents like ethanol (2 g/L) or DMSO (30 g/L). Studies on quercetin absorption performed in a rat in situ intestinal perfusion system found that ~9% of the applied dose was absorbed but due to a high secretion rate, only ~2% could be available for the distribution to peripheral tissues [89,90]. Quercetin metabolism is complex and involves intestinal uptake and further hepatic biotransformation. These modifications include deglycosylation, glucuronidation, sulfation, and methylation depending on the primary quercetin source. More polar metabolites are the result of these extensive modifications, which enhance quercetin elimination. It has recently been demonstrated that quercetin 3-O-β-d-glucuronide (Q3GA) and quercetin-3′-sulfate are the predominant quercetin conjugates in human plasma generated following its biotransformation [91,92]. The plasma concentration of quercetin in humans upon supplementation of this flavonoid could reach between the high nanomolar and the low micromolar range. The absorption of quercetin is related to its chemical nature. The aglycone is absorbed in the stomach and small intestine. While the absorption of quercetin glycosides occurs primarily in the small intestine after their cleavage to the aglycone form. The mechanism related to the intracellular transport of quercetin is associated with the organic anion transporting polypeptide (OATP) and it also occurs through passive diffusion [93,94]. Importantly, quercetin can cross the blood–retina barrier and reach the eye to provide therapeutic effects in ocular defects. In early studies on quercetin tissue distribution, quercetin was detected in isolated bovine retinas at a concentration of 40–70 ng/g of wet weight retina [95]. More recently, we have also detected quercetin in mouse eyes at a concentration of ~30 pmole/eye [96]. Nevertheless, the bioavailability of quercetin in the ocular tissue can be enriched thanks to the progress in the development of drug delivery systems. Using natural polymers, synthetic polymers and polymeric micelle-based nanoparticles loaded with quercetin enhanced its bioavailability in the eye, which was associated with the improved anti-inflammatory and antioxidant effects of quercetin [97,98,99,100]. Similar nano-formulations used previously for other drug delivery to the eye successfully increased the drug concentration within the eye and provided a platform for sustained drug delivery with advantageous biological effects [101,102]. Nevertheless, according to Lipinski’s rule of five, quercetin is compliant with druglike properties.

#### 5.1.3. Neuroprotective Biological Activities of Quercetin

Quercetin exhibits various biological activities, including antioxidant, anti-inflammatory, and anti-apoptotic effects with noted beneficial outcomes in various neurodegenerative diseases, including retinal degeneration [103,104].

##### Quercetin as an Antioxidant Agent

Free radicals produced by the body during metabolism can induce oxidative damage in biomolecules, such as carbohydrates, proteins, lipids, and nucleic acids [105]. Oxidative stress is an important factor contributing to the progression of photoreceptor death in RP regardless of the underlying genetic cause [62,106]. Thus, finding effective compounds with antioxidant properties to slow down pathological changes in RP has received researchers’ attention. Many antioxidants, including α-tocopherol, ascorbic acid, docosahexaenoic acid (DHA), and others that have been used in mouse models of RP, have demonstrated delayed degeneration of rods and increased survival of cone photoreceptors [107,108]. Among natural compounds, the neuroprotective effects of quercetin have been most investigated in various models of neurodegeneration, including RP [88,109,110]. Quercetin has been described as one of the most effective free radical scavengers, including O_2_^−^ and ONOO^−^, in the flavonoid family [35,111]. It was found that there are four hydroxyl groups on the benzo-dihydropyran ring of the polyphenol forming the pharmacophore of quercetin related to its strong antioxidant capacity. The antioxidant mechanisms of quercetin mainly include the following: directly scavenging free radicals, chelating metal ions, and modulating the expression of antioxidant enzymes [112,113]. These properties make quercetin a good inhibitor of lipid peroxidation, common in neurodegenerative diseases [90,114]. In addition, quercetin not only stops the propagation of lipid peroxidation but also increases GHS levels contributing to preventing free radical formation [115,116]. The antioxidant mechanisms of quercetin in vivo depend on the concentration of quercetin. Quercetin can directly scavenge ROS in vitro at concentrations of 5–50 µm [117]. However, it is unlikely that such high levels can be achieved in vivo in the peripheral tissues. In fact, as we recently found, quercetin can be detected in the mouse eye in a picomolar concentration [96]. In addition, although quercetin showed a protective effect against light-induced degeneration in mice susceptible to bright light injury, scavenging ROS by quercetin in the eyes of these mice was not effective [96]. Thus, the neuroprotective outcome of quercetin was rather related to the modulation of the cellular antioxidant defense mechanism, including SOD, catalase, and GSH peroxidase [118,119]. However, in RP-linked rd10 mice, treatment with quercetin at 100 mg/kg/day between P18 and P45 days of age resulted in a reduction of ROS levels with consequently enhanced survival of cone photoreceptors and improved the retinal function [88]. Unexpectedly, the expression of detoxifying enzymes such as SOD1 and 2 was diminished by prolonged quercetin administration, suggesting that the beneficial effect of this flavonoid can be related to the improvement of metabolic processes in photoreceptors that prevented oxidative stress and ROS generation. Moreover, it has been noted that quercetin, as well as other flavonoids, can counteract the oxidative insult by inducing the nuclear erythroid-derived factor 2 (Nrf2-ARE) pathway that plays an important role in anti-oxidative stress cellular defense [120,121]. Activation of this pathway provides neuroprotection against oxidative injury.

##### Quercetin as an Anti-Inflammatory Agent

**Figure 2:**
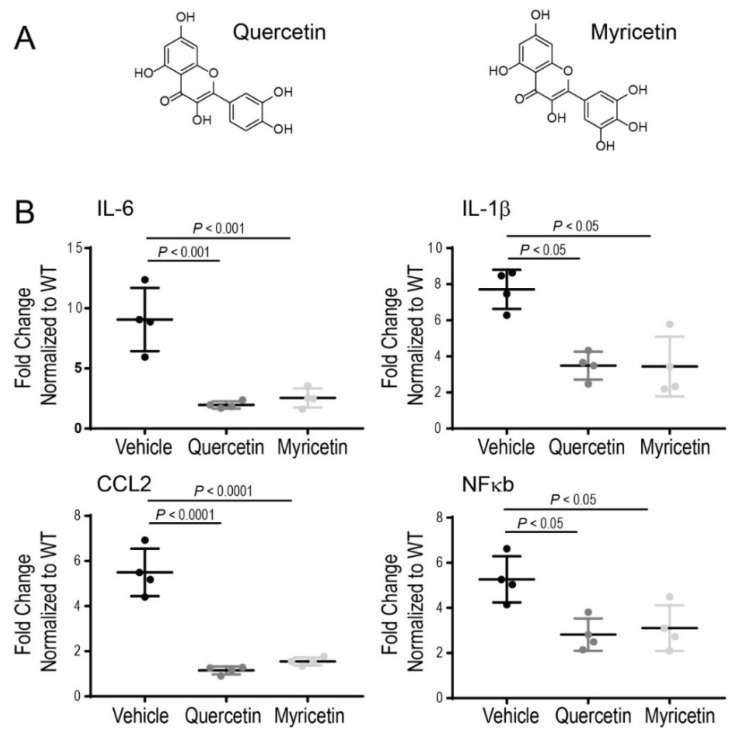
Regulation of the inflammatory markers by flavonoids in the homozygous P23H rhodopsin knock-in mice. (**A**) Chemical structures of two flavonoids, quercetin and myricetin. (**B**) The expression levels of selected inflammatory-response-related genes were examined by RT-qPCR. Mice were treated with quercetin or myricetin from postnatal (P) day 14 to P21 via intraperitoneal injection every other day at a dose of 20 mg/kg body weight. At P21 eyes were collected, and total RNA was isolated from the eyes of mice treated with flavonoids or a vehicle. Five mice were used for each treatment group. The Felative fold change of these genes’ expression was normalized to the expression of *Gapdh*. Error bars indicate standard deviation. The change in the expression of the analyzed genes was significantly reduced upon treatment with both flavonoids. The *p*-values for statistically different changes are indicated in the figure. Statistical analysis was performed using one-way ANOVA analysis and Bonferroni post hoc tests.

Recent in vitro and in vivo studies have demonstrated the anti-inflammatory effects of quercetin through the inhibition of pro-inflammatory markers with advantageous effects on cellular health [122,123]. Therefore, anti-inflammatory compounds could be beneficial in controlling the inflammatory process occurring under chronic disease conditions. It was reported previously that quercetin could inhibit the release of pro-inflammatory cytokines from the LPS-induced microglial cell line [124]. Moreover, inflammatory markers such as TNF-α, PGE2, and nitric oxide were substantially reduced in rat eyes with LPS-induced inflammation upon treatment with quercetin [125]. We have shown recently that quercetin can inhibit inflammatory reactions in mice acutely injured with bright light by suppressing the expression of pro-inflammatory molecules, including CCL2, IL-6, and TNF-α, which are implicated in degenerative processes in the retina under this condition [95]. Upregulation of pro-inflammatory cytokines such as TNF-α, IL-6, and IL-1β was also recently reported in the retinas of RP-related rd10 mice [108]. Treatment of these mice with a mixture of various nutraceuticals decreased the expression of these cytokines, resulting in delayed loss of photoreceptors and preserved function of the retina. To clarify further the anti-inflammatory effect of quercetin in RP we examined changes in the expression of several inflammatory markers, including IL-1β, IL-6, CCL2, and NFκb in the eyes of P23H rhodopsin knock-in mice by using RT-qPCR analysis. Quercetin was administered intraperitoneally to the homozygous P23H rhodopsin mice between P14 and P21 three times every other day at a dose of 20 mg/kg body weight. Upon treatment with quercetin, the expression of the above-mentioned markers was substantially decreased, indicating that quercetin can tune down inflammatory responses activated by the chronic insult related to the aberrant rhodopsin (Figure 2). These changes in the inflammatory markers correlated with improvements in retina morphology and function in these mice (manuscript under revision). A similar beneficial effect decreasing inflammatory markers in the homozygous P23H rhodopsin mice was found for another flavonoid myricetin administered to these mice using the same treatment scheme as for quercetin (Figure 2).

Beneficial health-improving effects of quercetin have been also noted in chronic inflammatory conditions other than neuroinflammation. In the gastrointestinal tract, treatment with quercetin attenuated experimental colitis and protected against experimental reflux esophagitis [126,127]. Quercetin can also decrease inflammatory responses in collagen-induced arthritis in mice [128].

Although further studies are necessary to validate the therapeutic use of quercetin in inflammatory-related diseases such as RP, considering the reported promising effects and the positive safety profile of this flavonoid should warrant controlled human clinical trials in the near future.

##### Quercetin as an Anti-Apoptotic Agent

Apoptosis is the most common mechanism of photoreceptor death in retinal degeneration. In response to the pathogenic stimulus, intrinsic apoptotic signaling is induced [129,130]. This pathway is related to the activation of pro-apoptotic protein Bax, which translocates from the cytoplasm to the outer mitochondrial membrane, where it forms a pore. Through such pores, cytochrome c is released from the mitochondria to the cytoplasm where it binds apoptotic protease factor 1 (Apaf-1) and ATP to activate pro-caspase 9, which then triggers the activation of the caspase cascade with caspase 3 considered as the main effector caspase. Caspase 3 proteolytically degrades the intracellular proteins leading to cell death [131]. Polyphenolic compounds including quercetin decrease membrane peroxidation and cytochrome c release, preventing the activation of caspase-mediated apoptosis [132]. The levels of Bax are controlled by the BcL-2 pro-survival protein and the ratio between these two proteins determines whether a cell lives or dies. Bax can also be activated by phosphoinositol-3-kinase (PI3K)/Akt signaling [133]. Quercetin can inhibit apoptosis through inhibition of the related signaling pathways. As recently shown, the PI3K/Akt pathway is involved in the apoptosis of photoreceptors in NaIO_3_-induced retinal degeneration. Interestingly, treatment with quercetin resulted in the significant improvement of retinal morphology in mice injured with NaIO_3_ [134]. A mechanistic study performed in ARPE-19 retinal cells revealed that quercetin likely inhibits the PI3K/Akt signaling pathway through the activation of BcL2, which inhibits Bax-mediated apoptosis [135]. As we found recently, the death of photoreceptors is mediated by Bax activation in mice acutely injured with bright light [96]. However, treatment with quercetin resulted in the inhibition of Bax expression and enhanced the retina levels of BcL-2, which correlated with the improved retina morphology and function in these mice [96]. Activation of pro-apoptotic Bax was also found in three models of RP, namely rd1, rhodopsin knockout, and transgenic P23H rhodopsin mice [136]. In addition to the modulation of Bax expression levels by quercetin, it is possible that quercetin directly binds to and inhibits Bax in a similar fashion as another flavonoid, icariin. It has been shown that icariin targets Bax, specifically blocking Bax dimer formation and its migration to the mitochondrial membrane [137]. The inhibition of Bax-dependent apoptosis by quercetin could also be related to the activation of sirtuins. Sirtuins are signaling proteins involved in the regulation of various cellular processes and metabolic pathways. Their actions can be modulated by flavonoids [138]. It has been shown that sirtuin (SIRT)1 protects neuronal cells from apoptosis through its involvement in DNA repair and regulating metabolic processes. SIRT1 also promotes the differentiation of stem cells. In rd10 mice the expression of SIRT1 was found in the outer nuclear layer and its expression pattern correlated with the beginning of retinal degeneration in these mice. Thus, it was concluded that SIRT1 is activated in the early stages of retinal degeneration to contribute to reparative processes [139]. However, as noted, the expression of SIRT1 may weaken over time.

Apoptotic processes are also regulated by calcium ion concentration. Increased levels of Ca^2+^ trigger photoreceptor death via caspase-dependent mechanisms. In photoreceptors isolated from rd1 mice an increase in Ca^2+^ concentration correlated with the elevation of calpain and triggered activation of caspase 12. The involvement of calpain 1 and cathepsin D in the regulation of apoptosis was also shown in rhodopsin knockout and P23H rhodopsin knock-in mice [136,140]. Inhibition of calpains with peptide inhibitors decreased the activity of caspase 7, resulting in improved photoreceptor survival [141].

## 6. Concluding Remarks

**Figure 3:**
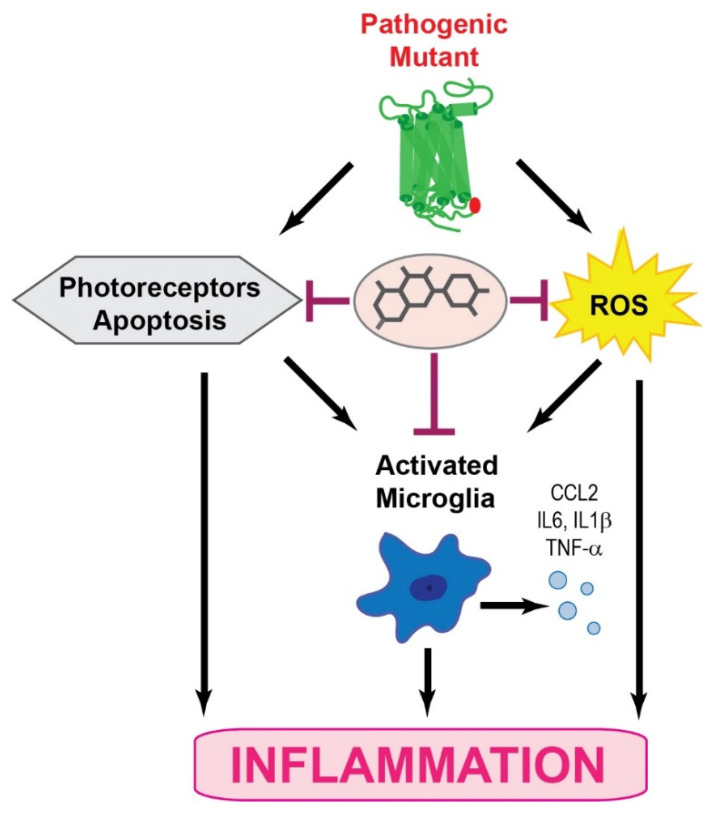
Prevention of RP retinal damage by flavonoids. The amino acid substitution of Pro23 to His in rhodopsin results in a structurally unstable receptor prone to aggregation in the endoplasmic reticulum ER, which induces the unfolded protein response (UPR) signaling and triggers the generation of reactive oxygen species (ROS). In addition, continuous stress caused by the pathogenic mutation leads to the activation of other cellular factors such as the microglia. Dysregulated microglia activation under sustained stressors enhances the inflammatory response, triggering the activation of the apoptotic process in the photoreceptor cells and RPE. Treatment with quercetin stabilizes the pathogenic rhodopsin mutant, which inhibits ROS generation, attenuates microglia activation, and slows down photoreceptor degeneration.

Chronic inflammation is a secondary effect of the genetic insult in RP and is considered to be one of the major player in the pathology of this visual impairment. The microglia are key cellular components associated with the response in this inflammatory process. Microglia are part of the immune cells implicated in neuronal homeostasis and innate immune response. In the early stages of RP, the microglia produce a neuroprotective or neurotrophic effect, which under prolonged insult is switched to a neurotoxic effect, aggravating inflammatory response through increased production of pro-inflammatory cytokines and chemokines. This change in the microglia activity is crucial to the development of retinal degeneration in RP. Pharmacological modulation of the microglia to shift their neurotoxic activities to neuroprotective effects in the retina could be an attractive therapeutic target. In addition, controlling the upregulation of pro-inflammatory cytokines could block cell transformation into neurotoxic states. Furthermore, the inflammatory response in retinal degenerative diseases suggests common pathways that could potentially be targeted in the development of therapeutic strategies combating RP. Thus, anti-inflammatory drugs hold great promise against retinal degeneration in RP. Natural products such as flavonoids could also serve as model molecules for the discovery of novel therapeutic avenues. In this regard, quercetin acts as a positive modulator of rod opsin and decreases levels of pro-inflammatory molecules in degenerating retinas with beneficial effects on retinal health (Figure 3). Thus, future studies evaluating the effects of quercetin in single or combinatory therapies with other anti-inflammatory drugs could result in developing treatment strategies for retinal degeneration. Although inflammation seems to be secondary in retinal degeneration, it is perhaps an important disease modifier. Thus, anti-inflammatory therapies could slow retinal degeneration, having a great impact on the life quality of affected individuals.

## Data Availability

The data presented in Figure 2 will be available upon reasonable request from the corresponding author.

## Abbreviations

AMD	Age-related macular degeneration
ATF6	Activating transcription factor 6
CC	Chemokine
COX	Cyclooxygenase
ER	Endoplasmic reticulum
GSH	Glutathione
IL	Interleukin
IRE1α	Inositol-requiring protein-1α
LPS	Lipopolysaccharide
MyD88	Myeloid differentiation factor
NLRP3	NOD-like receptor protein 3
PERK	Protein kinase RNA (PKR)-like ER kinase
PGE	Prostaglandin E
Rd	Retina degeneration
RP	Retinitis pigmentosa
RPE	Retina pigment epithelium
ROS	Reactive oxygen species
SOD	Superoxide dismutase
TNF	Tumor necrosis factor
TLR	Toll-like receptor
UPR	Unfolded protein response
